# The association of meal-specific food-based dietary inflammatory index with cardiovascular risk factors and inflammation in a sample of Iranian adults

**DOI:** 10.1186/s12902-023-01265-x

**Published:** 2023-01-10

**Authors:** Amin Mirrafiei, Shakila Ansari, Ahmad Jayedi, Azadeh Lesani, Kurosh Djafarian, Sakineh Shab-Bidar

**Affiliations:** 1 Department of Community Nutrition, School of Nutritional Sciences and Dietetics, Tehran University of Medical Sciences (TUMS), No 44, Hojjat-Dost Alley, Naderi St., Keshavarz Blvd, P. O. Box 14155/6117, Tehran, 14167-53955 Iran; 2 Department of Community Nutrition, School of Nutrition and Food Science, Isfahan University of Medical Sciences, Isfahan, Iran; 3 Department of Clinical Nutrition, School of Nutritional Sciences and Dietetics, Tehran University of Medical Sciences (TUMS), Tehran, 14167-53955 Iran

**Keywords:** Dietary inflammatory index, Meals, Cardiovascular disease, Insulin resistance, Inflammation

## Abstract

**Background:**

This study aimed to evaluate the association of meals-specific food-based dietary inflammatory index (FDII), with cardiovascular (CVD) risk factors and inflammation among Iranian adults.

**Methods:**

In this cross-sectional study, we recruited 816 participants living in Tehran via two-staged cluster sampling. Three non-consecutive 24-h dietary recalls (two working days and one day off) were obtained from individuals to specify the main meals and meal-specific FDIIs. Anthropometric measures were done. Insulin and high-sensitivity c-reactive protein (hs-CRP) were measured. Multiple linear regressions were used to investigate the association of FDII with Homeostatic Model Assessment for Insulin Resistance (HOMA-IR), hs-CRP, Triglyceride Glucose Index (TyG), and Lipid Accumulation Product Index (LAP).

**Results:**

The range of FDIIs for breakfast, lunch, and dinner were (-2.47,1.98), (-2.66,3.23) and (-4.09,3.13) in order, and the mean age was 42.2 ± 10.5 years. We found that there was no significant association between FDII and hs-CRP level in the three meals (β = -0.003; 95% CI: -0.030, 0.025 for breakfast,β = -0.020; 95% CI: -0.041, 0.001 for lunch, and β = 0.006; 95% CI: -0.016, 0.028 for dinner) after adjusting for age, sex, education, occupation, maritage, physical activity, smoking, morningness-eveningness score, energy, body mass index, and other FDIIs. Also, we found no significant association between breakfast, lunch, and dinner-specific FDII and HOMA-IR (β = -0.368, -0.223, 0.122), TyG index (β = -0.009, 0.060, -0.057) and LAP (β = 2.320, -0.278, -0.297).

**Conclusions:**

We found no associations between meal-based FDII scores and CVD and inflammation. Further research of prospective nature is needed to confirm these findings.

## Introduction

Inflammation is the body's natural biological response to physiological or metabolic stress caused by external or internal stimulation [1]. The histamine generated by damaged mast cells causes increased blood flow following tissue injury, which causes chronic inflammation [2]. Inflammation is recognized to play a significant role in the development of cardiovascular diseases (CVD) and related mortality [3]. CVD is considered the main cause of global death according to data from the World Health Organization (WHO) and kills about 18 million people annually, more than 30% of all mortalities [4]. Low-grade chronic inflammation can also increase the risk of atherosclerosis and insulin resistance (IR) [5]. IR is clinically defined as the inability of a person's body to provide enough insulin endogenously or exogenously to uptake and utilize available glucose [6]. It is believed to be a fundamental part of developing CVD since IR can lead to chronic hyperglycemia, which in turn causes an imbalance in glucose metabolism, oxidative stress, and an inflammatory response that damage cells [7].

Diet modification is a simple and influential method regarding chronic diseases [8]. The use of dietary indices and indicators that target dietary patterns and food combinations can be a more reliable predictor of non-communicable diseases. In addition, they are more straightforward to understand for the non-educated population [9]. Numerous studies explored the effect of modified diets or food patterns on inflammation [10–12]. Until recently, no tool was developed that could determine the inflammatory potential of foods. This led to the incarnation of the Dietary Inflammatory Index (DII) [13], which also made way for other indices including Empirical Dietary Inflammatory Pattern (EDIP) that assess the inflammatory potential of the diet based on the intake of food groups [14]. DII is an indicator designed by Shivappa et al. based on the antioxidant and oxidant properties of foods and has yielded positive results in detecting the inflammatory potential of foods [13, 15–17]. It was developed to provide a quantitative means for assessing the impact of diet on health outcomes spanning from concentrations of inflammatory cytokines in the serum to chronic diseases. According to a range from maximally pro-inflammatory to maximally anti-inflammatory, the DII classifies a person's diet based on how much inflammation it can cause. An inflammatory diet has a higher DII score, while an inflammatory diet has a lower DII score [3].

Many dietary assessment tools can be used to compute the DII, including 24-h dietary recall, which is the basis of FDII, the “food group based” dietary inflammatory index. The association of DII with CVD in different populations has previously been investigated [18]. A prospective study on high-risk participants showed that a pro-inflammatory diet indicated by a high DII score is directly linked to CVD [19]. In addition, a high DII score based on a one-time 24-h dietary recall was associated with a more inflammatory profile in Chinese patients [20]. Although the summary of results shows a positive relationship between the DII score and the risk of CVD, in most of the previous studies, the participants had a high level of education which could affect the type of diet. Also, in many studies, the status of blood lipids, one of the main risk factors, was not examined. On the other hand, the examination of food intake using a food frequency questionnaire (FFQ) based on long-term memory and also the small number of cases can cause the previous findings to not always be confirmed. It also seems that the type of food consumed by people usually does not change in the long term, which makes 24-h recall more appropriate and accurate. Furthermore, since the mixture of foods and drinks in the meals affects the whole diet, applying indices exclusively to the meals may embody a more understandable approach to dietary guidelines [21].

To the best of our knowledge, no research has been conducted to examine the association between the FDII of each of the three main meals of the day, with CVD and inflammation mutually, so we decided to do research on this purpose.

## Methods

### Participants

A sample of 816 healthy men and women, aged 20 to 59 years, who referred to health centers affiliated with Tehran University of Medical Sciences in 2018 and 2019, and were eager to participate were enrolled in the present cross-sectional study. Adults with a record of diabetes and cancer due to a possible change to their typical diet were excluded. A multi-stage cluster random sampling method was used to select health centers from the five districts (north, south, west, east, and center) of Tehran. Then, eight centers were chosen randomly from each district (*n* = 40). Lastly, by dividing the sample size by the total quantity of healthcare centers, the number of participants in each center was determined.

The sample collection was aided by the coordination of the Health Bureau of the Municipality of Tehran and the collaboration of the health care centers of Tehran. The study was conducted according to the guidelines laid down in the Declaration of Helsinki and all procedures involving human subjects/patients were approved by the ethical committee of the Tehran University of Medical Sciences (Ethic Number: IR.TUMS.VCR.REC.1400.2.212.52615). All patients received written information about the setting and methods of the study and signed a written informed consent form at the beginning of the study and received a copy of their reports.

### Demographic variables

Using the demographic questionnaire, we collected general information including the age, sex (female percentage), education (under diploma, diploma), marriage (married percentage), education (college-educated percentage), and smoking status (smoker percentage) of the participants.

### Physical activity

Physical activity was evaluated by employing the International Physical Activity Questionnaire (IPAQ) [22]. Based on the criteria, data were obtained about walking, moderate, and vigorous activity, in the previous week. Also, the time and frequency of activity days were recorded, and eventually, a physical activity score was calculated. The short form of the IPAQ, which records 3 intensity levels of activity based on the metabolic equivalents (METs) was used in the current study. METs were categorized as low (< 600 MET-minutes/week), moderate (600–3,000 MET-minutes/week), and vigorous (> 3,000 MET-minutes/week).

### MEQ score and sleep length

The Morningness-Eveningness Questionnaire (MEQ) was used to assess the circadian rhythm and sleep patterns of participants, based on 19 items on sleep habits and fatigue. Scoring was based on an original questionnaire by Östberg [23]. To estimate the mean sleep length of the individuals, sleep duration was obtained by using the short version of the Pittsburgh questionnaire, a self-reported questionnaire that assesses sleep quality and disturbances over a 1-month time interval, based on the 7 components: subjective sleep quality, sleep latency, sleep duration, habitual sleep efficiency, sleep disturbances, use of sleeping medication, and daytime dysfunction [24].

### Blood pressure and anthropometric measures

Participants' blood pressure was measured by a digital sphygmomanometer (BC 08, Beurer, Germany) after at least 10–15 min of rest and sitting. Blood pressure was measured twice per person and the average blood pressure per person was reported to report systolic and diastolic blood pressure (SBP and DBP), respectively. The height of patients was estimated by a wall gauge (stadiometer) with a sensitivity of 0.1 cm (Seca, Germany) without shoes, and weight was obtained with a digital scale (808 Seca, Germany) with an accuracy of 0.1 kg with light clothing (without coat and raincoat). Body mass index (BMI) was calculated by dividing weight in kilograms by height squared in meters. Waist circumference (WC) was measured with a tape measure between the lowest rib and the iliac crest when exhaling.

### Dietary assessment and meals definition

The dietary intake of the participants was collected employing three 24-h dietary recalls, as it supports a more comprehensive food preparation method explanation and clarifies the hour of consumption [25]. The first recall was asked by a trained interviewer on a face-to-face basis. Two other recalls were accumulated in non-consecutive days by phone calls. We obtained three main meals of the day by asking the amount and the daytime food intake, and the total and meal-specific energy per kcal, and other dietary components per grams using the Nutritionist IV application based on the US Department of Agriculture’s national nutrient data bank. Meals typically include breakfast, lunch, dinner, and snacks. If two or more meals were reported in 59 min or less margin, they were considered one meal and were combined using the average serving time. Otherwise, meal times were coded as a meal if they contributed more to the total daily energy and other meals as a snack [26–28].

### Meal-specific FDII calculation

We utilized an adjusted method used by Salari-Moghaddamet al. to calculate energy-adjusted meal-based FDII [29]. For this purpose, we used our dataset of a sample of Iranian people aged range 18–70 years old, in which foods and food groups contributing to systemic inflammation were explored. In that study, low-grade systemic inflammation was evaluated by measuring circulating high-sensitive C-reactive protein (hs-CRP) concentrations. Dietary intake was evaluated by three 24-h dietary recalls and then, the mean daily intakes of 28 pre-defined food groups (12 anti-inflammatory foods including fruits, fruit juices, fish, poultry, cruciferous vegetables, yellow vegetables, green leafy vegetables, other vegetables, tomatoes, legumes, whole grains, and tea and 16 pro-inflammatory foods including processed meats, red meats, eggs, butter, dairy, coffee, potatoes, French fries, refined grains, pizza, snacks, mayonnaise, soft drinks, sweets and desserts, hydrogenated fats, and hydrogenated oils) were obtained. All food groups were controlled for total energy using the residual method [30]. Finally, the mean daily intakes were multiplied by their given factor loadings for each participant [29]. The overall FDII score for each participant was calculated by summing the scores for each food and food group. Finally, we divided the FDII score by 100 to reduce the magnitude of the score. A higher FDII score (more positive) indicates a more pro-inflammatory diet and a lower FDII score (more negative) indicates a less pro-inflammatory diet. A higher FDII score (more positive) indicates a more pro-inflammatory diet and a lower FDII score (more negative) indicates a less pro-inflammatory diet.

### Laboratory investigations

Ten ml of fasting blood was taken from all participants between 7–10 am. It was poured into acid-washed test tubes without anticoagulants to be centrifuged at 1500 g for 20 min after being kept at room temperature (RT) for 30 min for blood clot formation. The serums were poured into clean microtubes and stored in a freezer at -80° C until the test was performed. Serum glucose and lipids were measured using the enzymatic method, based on colorimetry, utilizing commercial kits (Pars Azmoun, Iran) with an automatic device (Selecta E, Vitalab, Netherlands). Serum insulin was measured using commercial kits by the RIA insulin radioimmunoassay method and Serum hs-CRP was evaluated by immunoturbidimetry. Fasting plasma glucose (FPG), total cholesterol (TC), triglycerides (TG), high-density lipoprotein cholesterol (HDL-c), low-density lipoprotein cholesterol (LDL-c), hs-CRP, and uric acid(UA) levels were collected from blood samples.

### Outcome indices

The TyG Index is a novel index that predicts metabolic disorders and CVD. The TyG index, while simple, has high sensitivity and specificity; The TyG index has been approved in terms of cheapness and availability in terms of clinical applications [31]. It is calculated as follows:

$$\mathrm{Fasting\;TG }\;\left[\mathrm{mg}/\mathrm{d}1\right] *\left(\mathrm{Glucose }\;\left[\mathrm{mg}/\mathrm{d}1\right]/2\right)$$

LAP is a risk factor indicator for CVD that has greater sensitivity and specificity compared to waist circumference [32]. LAP, based on the superiority of waist circumference and fasting triglycerides, is utilized as a simple indicator of high lipid accumulation in adults. According to Kahn's study, compared to body mass index, LAP may be better for identifying adults at cardiovascular risk [33, 34]. It is calculated separately for men and women as follows:

$$\mathrm{Men}: \left(\mathrm{WC}/65\right)*\mathrm{TG};\mathrm{Women}: \left(\mathrm{WC}/58\right) *\mathrm{TG}$$

The homeostasis model assessment-estimated insulin resistance (HOMA-IR), was used for the estimation of IR. It is calculated by multiplying fasting plasma insulin (FPI) by fasting plasma glucose (FPG), then dividing by the constant 22.5. In addition, The homeostasis model assessment-estimated insulin sensitivity (HOMA-IS), was also evaluated to assess insulin sensitivity [35, 36].

### Statistical analysis

Mean and standard deviation as well as mean and range were used to describe the data. The general characteristics of participants across tertiles of the FDII in each meal were compared using Chi-square for qualitative variables and analysis of variance (ANOVA) test for quantitative variables. The macronutrient intake, serum biomarkers, and anthropometric measures were also compared across tertiles of FDII using analysis of covariance (ANCOVA) after controlling for age, sex, education, occupation, marital status, physical activity, smoking status, MEQ score, energy intake, and BMI (except for itself). In the next step, multiple linear regression was performed to model the association of FDII scores with hs-CRP, TyG, HOMA-IR, HOMA-IS, LAP, Fasting insulin, FPG, UA, TG, TC, LDL, HDL, BMI, WC, SBP, DBP, considering the possible confounding effect of other variables. A partial R^2^ value was reported. All analyzes were conducted by SPSS 26.0 statistical software. A *p*-value less than 0.05 is considered significant.

## Results

A total number of 816 healthy individuals were enrolled as the study population. The mean (± SD) age of participants was 42.2 (± 10.5) years with a mean BMI of 27.2 (± 4.5) kg/m2 and a female percentage of 82.3%. The range of FDIIs for breakfast, lunch, and dinner were (-2.47,1.98), (-2.66,3.23), and (-4.09,3.13), and the mean FDII was -0.30 ± 0.58, 0.68 ± 0.77, 0.33 ± 0.76, respectively (p-trend = 0.15). Characteristics of the participants across tertiles of the FDII are presented in Table 1. There were no significant differences in the mean age, level of physical activity, marital status, mean MEQ score, and sleep length of participants based on meal-specific FDII tertiles. The percentage of smokers in the top tertile of the lunch FDII score was significantly lower than the bottom tertile (*p* = 0.01).

**Table 1:** Anthropometric measures and serum biomarkers of the study participants across tertiles of meal-specific FDII

	**Breakfast FDII (-0.30 ± 0.58)**			***P***	**Lunch FDII (0.68 ± 0.77)**			***P***	**Dinner FDII (0.33 ± 0.76)**			**P-trend = 0.15**
	T1	T2	T3		T1	T2	T3		T1	T2	T3	***P***
**Age (y)**	42.5 ± 10.4	42.2 ± 10.4	42.0 ± 11.0	0.86	41.1 ± 10.2	42.2 ± 10.4	43.1 ± 11.0	0.08	42.6 ± 10.6	42.9 ± 10.2	41.3 ± 10.9	0.17
**Women %**	83.1	81.6	82.4	0.90	82.1	83.2	81.7	0.89	84.2	81.2	80.8	0.55
**Smoking %**	1.5	4.0	4.0	0.19	5.5	1.8	2.6	0.01	2.6	3.0	4.5	0.55
**Married %**	81.3	81.6	77.9	0.44	80.6	82.8	79.1	0.32	80.4	84.2	78.2	0.18
**Physical activity %**												
Low	54.8	50.4	51.1	0.29	53.8	53.1	50.9	0.94	55.8	49.2	53.0	0.47
Moderate	37.5	37.9	41.5		37.7	38.5	39.2		35.5	40.6	39.8	
High	7.7	11.8	7.4		8.4	8.4	9.9		8.7	10.2	7.1	
**Higher education %**	36.8	30.9	37.9	0.28	35.9	36.3	31.1	0.54	31.7	36.1	34.6	0.75
**MEQ score**	59.1 ± 5.81	58.9 ± 5.72	58.0 ± 5.89	0.07	58.7 ± 6.14	58.5 ± 5.78	58.8 ± 5.63	0.79	58.7 ± 5.82	58.5 ± 5.93	58.7 ± 5.86	0.84
**Sleep length (h)**	5:54 ± 2:36	5:57 ± 2:27	6:19 ± 2:08	0.08	6:07 ± 2:20	6:02 ± 2:28	5:54 ± 2:32	0.61	6:04 ± 2:27	6:09 ± 2:26	5:57 ± 2:24	0.61
**FDII score**	-0.90 ± 0.28	-0.38 ± 0.17	0.38 ± 0.33	< 0.001	-0.12 ± 0.56	0.71 ± 0.15	1.46 ± 0.44	< 0.001	-0.47 ± 0.60	0.38 ± 0.15	1.08 ± 0.40	< 0.001

Data is mean and standard deviation unless reported

*MEQ* Morningness-Eveningness Questionnaire, *DII* Dietary inflammatory index, *T* Tertile

*P* < 0.05 is significant (analysis of variance or chi-square test was used)

The macronutrients and food groups intakes of the participants across tertiles of the FDII are indicated in Table 2. Participants with the highest FDII score for lunch had significantly greater intakes of carbohydrates and energy compared with those in the lowest tertile (*p* < 0.001). Also, we found that participants in the top tertile of FDII score had lower meal protein intake for lunch and dinner compared with the first tertile, in contrast to the breakfast where lower FDII score was associated with a lower protein intake (*p* = 0.01). The fat intake did not significantly differ across tertiles of any FDII score.

**Table 2:** Dietary intakes of the participants across tertiles of meal-specific FDII

	**Breakfast FDII**			***P***	**Lunch FDII**			***P***	**Dinner FDII**			***P***
	T1	T2	T3		T1	T2	T3		T1	T2	T3	
**Energy (Kcal/d)**	456.0 ± 14.7	438.9 ± 14.7	437.4 ± 14.7	0.61	551.5 ± 16.1	481.3 ± 16.0	578.6 ± 16.1	< 0.001	568.4 ± 17.4	434.2 ± 17.4	561.5 ± 17.4	< 0.001
**Protein (g/d)**	12.5 ± 0.33	12.5 ± 0.33	13.8 ± 0.33	0.01	25.5 ± 0.64	23.0 ± 0.64	19.7 ± 0.64	< 0.001	20.7 ± 0.50	19.5 ± 0.50	18.5 ± 0.49	0.01
**Carbohydrate (g/d)**	69.4 ± 1.72	71.6 ± 1.72	71.7 ± 1.72	0.56	61.3 ± 2.60	64.5 ± 2.61	75.7 ± 2.61	< 0.001	73.2 ± 13.7	68.4 ± 13.9	108.9 ± 13.7	0.07
**Meal fat (g/d)**	14.1 ± 2.41	17.9 ± 2.41	13.3 ± 2.41	0.35	21.1 ± 0.51	20.2 ± 0.51	20.7 ± 0.51	0.54	17.9 ± 0.56	18.4 ± 0.57	17.5 ± 0.56	0.52
**Vegetables (g/d)**	10.5 ± 2.21	7.43 ± 2.22	8.43 ± 2.21	0.59	93.6 ± 4.17	37.0 ± 4.17	20.3 ± 4.17	< 0.001	97.4 ± 4.31	33.2 ± 4.29	28.1 ± 4.27	< 0.001
**Fruits (g/d)**	6.02 ± 1.24	5.19 ± 1.24	1.46 ± 1.24	0.02	8.26 ± 1.21	1.59 ± 1.21	1.73 ± 1.21	< 0.001	13.0 ± 1.61	1.83 ± 1.60	1.00 ± 1.59	< 0.001
**Rice (g/d)**	0.00 ± 0.55	0.28 ± 0.55	2.37 ± 0.55	0.01	71.4 ± 4.79	111.7 ± 4.79	170.4 ± 4.79	< 0.001	22.3 ± 4.33	42.2 ± 4.31	114.3 ± 4.30	< 0.001
**Bread (g/d)**	16.5 ± 2.72	60.2 ± 2.72	56.5 ± 2.72	< 0.001	11.4 ± 3.85	18.0 ± 3.85	38.9 ± 3.85	< 0.001	16.0 ± 3.70	22.0 ± 3.68	47.6 ± 3.67	< 0.001
**Egg (g/d)**	2.24 ± 1.31	6.45 ± 1.31	13.6 ± 1.31	< 0.001	4.71 ± 1.08	4.39 ± 1.08	6.28 ± 1.08	0.41	7.72 ± 1.45	13.1 ± 1.44	12.3 ± 1.44	0.01
**Poultry (g/d)**	0.34 ± 0.31	0.63 ± 0.31	1.45 ± 0.31	0.01	35.9 ± 2.42	16.0 ± 2.42	10.3 ± 2.42	< 0.001	16.7 ± 1.79	10.5 ± 1.78	8.00 ± 1.78	0.01
**Red meat (g/d)**	0.00 ± 0.20	0.08 ± 0.20	0.34 ± 0.20	0.45	4.97 ± 1.11	6.53 ± 1.10	6.33 ± 1.11	0.55	0.96 ± 0.61	0.78 ± 0.60	4.61 ± 0.60	< 0.001
**Dairy (g/d)**	4.15 ± 3.00	8.92 ± 3.00	21.2 ± 3.00	< 0.001	42.2 ± 5.92	41.4 ± 5.91	42.5 ± 5.92	0.99	49.6 ± 5.70	42.8 ± 5.68	29.4 ± 5.66	0.04
**Legume (g/d)**	0.15 ± 0.35	0.70 ± 0.35	0.95 ± 0.35	0.25	16.9 ± 1.67	15.2 ± 1.67	16.3 ± 1.67	0.77	12.8 ± 1.61	8.35 ± 1.60	10.3 ± 1.59	0.14

Data is mean and Standard deviation; *g* grams, *T*, tertile

Obtained by ANCOVA, adjusted for age, sex, energy intake (except for itself)

*P* < 0.05 is significant

As reported in Table 3, despite adjustments for potential confounders, consisting of age, sex, education, occupation, marital status, physical activity, smoking status, MEQ score, energy intake, and BMI (except for itself), we did not observe any significant variations in the mean SBP, DBP, FPG, TG, HDL-c, LDL-c, TC, BMI, HOMA-IR, HOMA-IS, LAP, TyG and fasting insulin of participants comparing tertiles of FDII score. Although the mean UA in the top tertile of the FDII score was higher than the lowest tertile in all meal subgroups, this relationship was only significant in the dinner subgroup (*p* = 0.009). Interestingly, compared with the ones in the highest tertile, participants in the first tertile of FDII score had greater mean hs-CRP; however, this result turned into significant just for the lunch meal (*p* = 0.034).

**Table 3:** Values of anthropometric measures and blood factors of the study participants across tertiles of meal-specific FDII

	**Breakfast FDII**			***P***	**Lunch FDII**			***P***	**Dinner FDII**			***P***
	T1	T2	T3		T1	T2	T3		T1	T2	T3	
**BMI (kg/m**^**2**^**)**	27.2 ± 0.27	27.0 ± 0.28	27.1 ± 0.28	0.83	27.0 ± 0.28	27.3 ± 0.27	27.2 ± 0.27	0.82	27.5 ± 0.28	27.2 ± 0.27	26.9 ± 0.28	0.34
**Waist circumference (cm)**	88.5 ± 0.52	88.7 ± 0.52	89.4 ± 0.52	0.49	89.0 ± 0.53	89.5 ± 0.53	89.1 ± 0.52	0.78	88.8 ± 0.53	88.8 ± 0.52	88.9 ± 0.53	0.19
**SBP (mmHg)**	118.4 ± 0.86	117.7 ± 0.86	117.9 ± 0.87	0.86	116.8 ± 0.87	118.5 ± 0.87	118.7 ± 0.87	0.25	117.0 ± 0.88	117.7 ± 0.87	119.2 ± 0.88	0.20
**DBP (mmHg)**	78.7 ± 0.57	78.5 ± 0.57	78.8 ± 0.57	0.90	78.1 ± 0.58	79.2 ± 0.57	78.6 ± 0.57	0.37	78.5 ± 0.58	78.2 ± 0.57	79.2 ± 0.58	0.46
**LDL (mg/dl)**	115.3 ± 2.37	119.4 ± 2.37	119.0 ± 2.39	0.41	116.0 ± 2.40	120.6 ± 2.40	116.0 ± 2.39	0.29	120.8 ± 2.42	115.1 ± 2.40	116.8 ± 2.43	0.23
**HDL (mg/dl)**	49.8 ± 0.61	49.8 ± 0.61	50.3 ± 0.61	0.83	48.7 ± 0.60	49.8 ± 0.60	50.1 ± 0.59	0.24	50.4 ± 0.60	49.2 ± 0.60	49.1 ± 0.60	0.23
**TC (mg/dl)**	193.9 ± 2.72	195.6 ± 2.72	197.7 ± 2.74	0.62	192.9 ± 2.76	198.9 ± 2.75	193.0 ± 2.74	0.21	197.4 ± 2.78	192.3 ± 2.75	195.3 ± 2.78	0.38
**TG (mg/dl)**	146.3 ± 4.41	138.6 ± 4.41	144.8 ± 4.40	0.43	143.9 ± 4.40	149.3 ± 4.40	138.5 ± 4.38	0.22	138.8 ± 4.43	142.4 ± 4.38	150.6 ± 4.43	0.15
**Uric acid (mg/dl)**	4.66 ± 0.08	4.73 ± 0.07	4.68 ± 0.07	0.82	4.63 ± 0.07	4.74 ± 0.08	4.67 ± 0.08	0.60	4.57 ± 0.08	4.60 ± 0.07	4.88 ± 0.08	0.01
**Fasting Insulin (mIU/L)**	15.3 ± 0.80	13.8 ± 0.80	12.8 ± 0.81	0.09	13.8 ± 0.74	13.0 ± 0.74	13.6 ± 0.74	0.76	14.3 ± 0.75	13.2 ± 0.74	12.9 ± 0.75	0.35
**FPG (mg/dl)**	106.5 ± 1.19	103.2 ± 1.19	105.8 ± 1.20	0.11	105.2 ± 1.17	105.7 ± 1.17	103.6 ± 1.17	0.44	105.1 ± 1.18	103.5 ± 1.16	105.9 ± 1.18	0.34
**HOMA-IR**	4.17 ± 0.25	3.65 ± 0.26	3.38 ± 0.26	0.08	3.65 ± 0.23	3.44 ± 0.23	3.58 ± 0.22	0.81	3.84 ± 0.23	3.45 ± 0.22	3.39 ± 0.23	0.31
**HOMA-IS**	2.86 ± 0.14	2.66 ± 0.14	2.49 ± 0.14	0.16	2.65 ± 0.13	2.50 ± 0.13	2.65 ± 0.13	0.67	2.75 ± 0.13	2.54 ± 0.13	2.51 ± 0.13	0.40
**LAP**	48.9 ± 1.84	47.3 ± 1.84	51.0 ± 1.85	0.37	49.4 ± 1.85	51.4 ± 1.85	48.1 ± 1.84	0.45	48.2 ± 1.86	48.2 ± 1.84	52.7 ± 1.86	0.14
**TyG**	5.02 ± 0.18	4.65 ± 0.18	4.93 ± 0.18	0.31	4.89 ± 0.17	5.12 ± 0.17	4.61 ± 0.17	0.12	4.71 ± 0.18	4.75 ± 0.17	5.16 ± 0.18	0.14
**CRP (mg/dl)**	0.20 ± 0.01	0.21 ± 0.01	0.19 ± 0.01	0.79	0.22 ± 0.01	0.17 ± 0.01	0.20 ± 0.01	0.04	0.20 ± 0.01	0.20 ± 0.01	0.18 ± 0.01	0.43

*SBP* Systolic blood pressure, *DBP* Diastolic blood pressure, *FPG* Fasting plasma glucose, *TG* Triglyceride, *HDL* High-density lipoprotein, *LDL* Low-density lipoprotein, *TC* Total cholesterol, *BMI* Body mass index, *HOMA-IR* Homeostatic Model Assessment for Insulin Resistance, *HOMA-IS* Homeostatic Model Assessment for Insulin sensitivity, *LAP* Lipid accumulation product index, *TyG* Triglyceride-glucose index, *CRP* C-reactive protein, *T* tertile, *SE* Standard error

Obtained by ANCOVA, adjusted for age, sex, education, occupation, marital status, physical activity, smoking status, MEQ score, energy intake, and BMI (except for itself)

Values are mean ± standard error

*P* < 0.05 is significant

Multiple linear regression analysis of anthropometric measures, serum biomarkers, CVD risk factors, and meal-specific FDII is shown in Table 4. The breakfast-specific FDII had the strongest correlation with HOMA-IR (partial *R* = -0.053), LAP (partial *R* = 0.047), FPG (partial *R* = -0.012), and WC (partial *R* = 0.053), while hs-CRP (partial *R* = -0.070), TyG (partial *R* = 0.017), HOMA-IS (partial *R* = -0.071), fasting insulin (partial *R* = -0.058), TC (partial *R* = -0.056), LDL-c (partial *R* = -0.063), and DBP (partial *R* = 0.049) had the strongest correlation with the lunch-specific FDII. Finally, the degree of association was highest between dinner-specific FDII and UA (partial *R* = 0.069), TG (partial *R* = -0.015), HDL (partial *R* = 0.046), BMI (partial *R* = -0.048) and SBP (partial *R* = 0.055), after controlling the effect of confounders and other meal-specific FDIIs. A negative non-significant relationship was found between FDII score (breakfast and lunch) and hs-CRP, HOMA-IR, HOMA-IS, and fasting insulin. Unlike lunch, an increase in breakfast and dinner FDII scores was associated with an increase in blood UA, TC, LDL-c, and HDL-c; however, these relationships were not significant for breakfast FDII score (β = 0.047; 95% CI: -0.11, 0.203, β = 0.528; 95% CI: -4.765, 5.821, β = 0.194; 95% CI: -4.432, 4.819 and β = 0.024; 95% CI -1.159, 1.207 for breakfast FDII score respectively). We observed a non-significant positive association between SBP and DBP with all of the meal-specific FDII scores (β = 0.496; 95% CI: -0.757, 1.748, β = 0.566; 95% CI: -0.262, 1.395 for lunch FDII score) respectively.

**Table 4:** Multiple linear regression analysis of anthropometric measures and blood factors and meal specific dietary inflammatory indices

	R^2^		Breakfast FDII	Lunch FDII	Dinner FDII
HS-CRP	0.237	**β**	-0.003	-0.020	0.006
		**95% CI**	-0.030, 0.025	-0.041, 0.001	-0.016, 0.028
		**Partial R**	-0.007	-0.070	0.020
TyG	0.284	**β**	-0.009	0.060	-0.057
		**95% CI**	-0.353, 0.335	-0.198, 0.318	-0.330, 0.217
		**Partial R**	-0.002	0.017	-0.015
HOMA-IR	0.203	**β**	-0.368	-0.223	0.122
		**95% CI**	-0.866, 0.130	-0.597, 0.151	-0.274, 0.517
		**Partial R**	-0.053	-0.043	0.022
HOMA-IS	0.227	**β**	-0.146	-0.201	0.067
		**95% CI**	-0.417, 0.125	-0.404, 0.002	-0.148, 0.272
		**Partial R**	-0.039	-0.071	0.023
LAP	0.520	**β**	2.320	-0.278	-0.297
		**95% CI**	-1.247, 5.887	-2.955, 2.398	-3.127, 2.533
		**Partial R**	0.047	-0.008	-0.008
Fasting insulin	0.215	**β**	-1.069	-0.950	0.380
		**95% CI**	-2.634, 0.495	-2.124, 0.223	-0.861, 1.621
		**Partial R**	-0.049	-0.058	0.022
FPG	0.262	**β**	-0.388	-0.049	-0.204
		**95% CI**	-2.707, 1.931	-1.789, 1.690	-2.044, 1.635
		**Partial R**	-0.012	-0.002	-0.008
UA	0.355	**β**	0.047	-0.022	0.119
		**95% CI**	-0.110, 0.203	-0.139, 0.096	-0.005, 0.243
		**Partial R**	0.022	-0.013	0.069
TG	0.256	**β**	1.119	-0.328	-1.382
		**95% CI**	-7.436, 9.673	-6.746, 6.090	-8.169, 5.405
		**Partial R**	0.009	-0.004	-0.015
TC	0.266	**β**	0.528	-3.090	0.211
		**95% CI**	-4.765, 5.821	-7.061, 0.881	-3.989, 4.410
		**Partial R**	0.007	-0.056	0.004
LDL	0.237	**β**	0.194	-3.013	0.063
		**95% CI**	-4.432, 4.819	-6.484 – 0.457	-3.607 – 3.733
		**Partial R**	0.003	-0.063	0.001
HDL	0.290	**β**	0.024	-0.002	0.593
		**95% CI**	-1.159, 1.207	-0.989, 0.886	-0.346, 1.532
		**Partial R**	0.001	< 0.001	0.046
BMI	0.292	**β**	0.044	0.091	-0.282
		**95% CI**	-0.494, 0.581	-0.312, 0.494	-0.708, 0.144
		**Partial R**	0.006	0.016	-0.048
WC	0.720	**β**	0.742	-0.170	0.310
		**95% CI**	-0.263, 1.747	-0.924, 0.584	-0.488, 1.107
		**Partial R**	0.053	-0.016	0.028
SBP	0.405	**β**	0.540	0.496	1.002
		**95% CI**	-1.130, 2.209	-0.757, 1.748	-0.322, 2.327
		**Partial R**	0.023	0.029	0.055
DBP	0.290	**β**	0.559	0.566	0.115
		**95% CI**	-0.546, 1.663	-0.262, 1.395	-0.762, 0.992
		**Partial R**	0.037	0.049	0.009

*SBP* Systolic blood pressure, *DBP* Diastolic blood pressure, *FPG* Fasting plasma glucose, *TG* Triglyceride, *HDL* High-density lipoprotein *LDL* Low-density lipoprotein, *TC* Total cholesterol, *WC* Waist circumference, *HC* Hip circumference, *BMI* Body mass index, *HOMA-IR* Homeostatic Model Assessment for Insulin Resistance, *HOMA-IS* Homeostatic Model Assessment for Insulin sensitivity, *LAP* Lipid accumulation product index, *TyG* Triglyceride-glucose index, *CRP* C-reactive protein

All adjusted for other FDIIs, age, sex, education status, occupation status, marital status, physical activity, smoking status, MEQ score, energy intake, and BMI (except for itself)

## Discussion

In this cross-sectional study, we found no significant association between meal-specific FDII and inflammation. Also, no significant association was observed between breakfast, lunch, and dinner-specific FDII and CVD risk factors including the lipid profile, UA concentrations, and anthropometric measures.

FDII is an apriori food-based inflammatory index, while DII is nutrient based. FDII can provide more complete detail about the impact of food on health outcomes rather than DII, as nutrients are consumed as a whole and have synergic effects on each other. In addition, a recommendation based on the food groups is more practical for the general population. Similar to FDII, EDIP, an a posteriori inflammatory index, measures the inflammatory potential of diet as the sum of the effects of pro- and anti-inflammatory foods in complete diets and is based solely on food groupings [37]. FDII can be considered a summary of both DII and EDIP. In line with our findings, in a prospective cohort study conducted by Neufcourt et al. on French adults, a pro-inflammatory diet, measured by the DII, was not significantly associated with the risk of CVD including angina pectoris and stroke [38]. Another cohort study on 6972 middle-aged Australian women by Phillips et al. showed no significant association between DII and the risk of CVD, ischemic heart disease, myocardial infarction, cerebrovascular disease, and stroke [39]. In addition, a nested cross-sectional study by Carvalho et al. reported no association between the increase in DII score and IR. the results were similar after adjusting for some confounders in both men and women subgroups [40]. Inflammatory cytokines that suppress insulin response in adipose tissue, skeletal muscle, and liver such as interleukin-1beta (IL-1β) and interleukin-6 (IL-6), are known to be parts of mechanisms that link pro-inflammatory conditions to IR [41]. Hyperglycemia caused by IR can have detrimental effects on endothelium, vascular smooth muscle cells, and macrophages, causing thrombosis and fibrinolysis leading to the formation of atherosclerotic plaques. Furthermore, the overproduction of reactive oxygen species and advanced glycation end-products further increases low-grade inflammation, contributing to increased CVD risk [42]. Garcia-Arellano et al. in a prospective study (Prevención con Dieta Mediterránea) on high-risk participants revealed that a pro-inflammatory diet assessed by a 137-item FFQ is directly associated with clinical CVD events [19]. Another cohort study by Ramallal et al. on a large population of educated middle-aged Spanish adults showed that a higher DII score is directly associated with CVD risk factors [43]. A cross-sectional study by Kim et al. also revealed that being in the 4th quartile of DII made men 1.3 times more likely to develop hyperglycemia but it turned into non-significant when assessed in the women subgroup [44]. In another cohort study conducted by Farhadnejad et al. The higher inflammatory potential of diet assessed by dietary inflammation score was associated with a higher risk of IR in Tehranian adults [45]. It is worth noting that all these studies have used the original DII rather than FDII; therefore, our findings might not be able to compare our results, as the synergic effects are somehow hidden in the nutrient-based model.

CRP is an indicator of chronic inflammation which can raise in many disorders such as diabetes, CVD, and metabolic syndrome [46–48]. In this study, meal-specific FDII was not related to hs-CRP concentration. In contrast, to our findings, a recent study by Kotemori et al. which examined the validity of DII score by assessing hs-CRP levels in a sample of Japanese men and women declared that men in the top quartile of DII score were 1.72 times more likely to have hs-CRP concentration > 3 mg/L, but no association was observed among women [49]. Moreover, a prospective cohort of 6000 adults by Cavicchia et al. similarly showed that an anti-inflammatory diet was associated with a reduced likelihood of high hs-CRP levels [50]. Philips et al., in a cross-sectional study, obtained the dietary intake of the 1992 participants using a validated FFQ to explore the association of its driven DII with biomarkers of lipoprotein metabolism, inflammation, and glucose homeostasis. The results of the study revealed that a more pro-inflammatory diet is linked to an increase in the concentration of LDL, and inflammation, characterized by a higher level of CRP, by using energy-adjusted nutrient-based DII [17]. In another study, Ren et al. assessed the association between DII score, based on one-time 24-h dietary recall, CRP, and metabolic syndrome. The authors found that a high score of DII is associated with a higher number of CRP components in the second and third tertile of Chinese patients with metabolic syndrome [20]. Shivappa et al. evaluated the predictive ability of 24-h recall-derived DII on inflammation among 532 European adolescents in the HELENA cross-sectional study. They found that a pro-inflammatory diet (higher DII scores) was associated with increased levels of inflammatory markers after adjusting for different confounders [51]. The lack of significant association between FDII and inflammation in our study may be related to the fact that stress and fatigue also can contribute to an increase in the concentration of hs-CRP [52]. In this study, participants were healthy adults with a normal intake of fat and carbohydrate in each specific meal, also the mean sleep length seemed to be adequate, and less than 10% of individuals in each tertile of FDII score were smokers.

Noteworthy, the means of the FDII score of breakfast, lunch, and dinner in this study were -0.30, 0.68, and, 0.33, respectively, which shows an increasing trend, but does not characterize a pro-inflammatory diet in general. In addition, the nature of the Iranian diet is less inflammatory than the Western diet [53, 54] Participants with the highest tertile of FDII in all meals had higher consumption of rice, bread, and poultry and a lower intake of vegetables and fruits. In eastern Asian studies consumption of higher rice was not associated with higher hs-CRP levels as Zuniga et al. demonstrated that rice and noodle consumption was not associated with hs-CRP concentrations and also suggested that high consumption of refined grains may contribute to hyperglycemia through greater IR, rather than through increased systemic inflammation [55]. A higher intake of red meat is supposed to lead to elevated inflammation biomarkers as Ley et al. concluded that greater red meat intake was in line with undesirable plasma concentrations of inflammatory and glucose metabolic biomarkers in non-diabetic women [56]. The average intake of red meat in any of the FDII tertiles of this study was not higher than 7 g, which was very low compared to the average intake of red meat in other studies, this could be a possible reason for the non-significant association that observed in this study.

To the best of our knowledge, this is the first study that investigated the relationship between FDII and inflammation and CVD biomarkers based on meals. Traditionally, most of the studies focused on the relationship between habitual intake and health conditions [57]. Studies on meal patterns can give us information about meal frequency, spacing, skipping, and timing [58], and show how different combinations of foods and beverages at eating events could inspire the overall diet quality. Meals-based advice might be easier and more practical for the population to comprehend and apply to their diets [59].

This study has several strengths; first, the study has a large sample size. Second, participants were recruited from different parts of the city of Tehran, which helps with the generalization of the results. Third, this is the first study that evaluates the FDII score for the main three dietary meals. The type of food consumed by people usually does not alter in the long term, which makes 24-h recall more accurate. Furthermore, we additionally adjusted for MEQ scores as confounding variables. In some evidence, the duration of sleep may be associated with the risk of CVD outcomes, and long sleep was correlated with systemic inflammation [60, 61]. In addition, late eating, which is closely associated with MEQ scores, affects the pattern of dietary intake such as low breakfast consumption rate and eating of unhealthy foods with pro-inflammatory effects, in advance leading to CVD [62]. Despite all strengths, some limitations should also be addressed. First, to obtain more reliable results, the proportion of women to men should be close to 1, but in this study, the number of men was considerably lower than women. Secondly, due to the cross-sectional nature of this study, the causal relationship between FDII score and CVD and inflammation risk factors cannot truly be inferred. In addition, measurement errors and misclassification of study participants across the tertiles of FDII score could not be avoided, but we tried our best to reduce the measurement error by collecting the data via a trained dietitian and considering the role of potential confounders in the data analysis.

## Conclusion

Overall, no significant association was found between meal-specific FDII scores and CVD risk factors and inflammation in the present study. Further research, particularly of prospective nature, is needed to confirm these findings.

## Data Availability

The datasets generated and/or analysed during the current study are not publicly available due privacy of the data but are available from the corresponding author on reasonable request.

## Abbreviations

FDII: Food-based Dietary Inflammatory Index
CVD: Cardiovascular disease
IR: Insulin Resistance
hs-CRP: High-sensetivity C-reactive Protein
BMI: Body Mass Index
WC: Waist Circumference
SBP: Systolic Blood Pressure
DBP: Diastolic Blood Pressure
LDL: Low-density Lippoprotein
HDL: High-density Lippoprotein
TC: Total Cholesterol
TG: Triglyceride
FPG: Fasting Plasma Glucose
HOMA-IR: Homeostatic Model Assessment for Insulin Resistance
HOMA-IS: Homeostatic Model Assessment for Insulin sensitivity
LAP: Lipid Acummulation Product
TyG: Triglyceride-Glucose Index
